# Questions regarding mortality and infection source in deep-seated infections

**DOI:** 10.1128/aac.01664-25

**Published:** 2026-03-02

**Authors:** Sho Nishimura

**Affiliations:** 1 Department of Infectious Disease, Hyogo Prefectural Harima-Himeji General Medical Center, Himeji, Japan; University of Fribourg, Fribourg, Switzerland

**Keywords:** mortality, deep-seated infection, deep-abscess

## LETTER

The study by Slain et al. (1) is particularly interesting as it presents results contrary to several previous observational studies suggesting that cefepime may be a suitable alternative to carbapenems. However, I still have some questions as to whether meropenem is truly superior to cefepime for deep-seated infections caused by chromosomally encoded AmpC-producing organisms at high risk for derepression.

First, why was the 30-day mortality in the cefepime group higher than the 60- or 90-day mortality rates? Even in the absence of statistical significance, it is somewhat puzzling that the cefepime group had numerically lower 60- and 90-day mortality and readmission rates, while only the primary outcome—clinical treatment failure—was higher in the cefepime group.

Additionally, there appear to be some differences in the site of infection between groups. Numerically, deep abscesses were more frequent in the cefepime group, but where exactly were these “deep abscesses” due to monomicrobial *Enterobacterales* infections located? In many infections involving *Enterobacterales*, coverage for other *Enterobacterales* or anaerobes would typically be required. Cefepime does not provide anaerobic coverage. Moreover, while most deep abscesses require drainage, certain bone infections, such as vertebral osteomyelitis can sometimes resolve without drainage. The actual number of cases where source control was necessary should be clarified, and among cases where source control was required, the frequency at which it was actually performed would be a more meaningful metric.
